# Curcumin protects rat liver from streptozotocin-induced diabetic pathophysiology by counteracting reactive oxygen species and inhibiting the activation of p53 and MAPKs mediated stress response pathways

**DOI:** 10.1016/j.toxrep.2014.12.017

**Published:** 2015-01-02

**Authors:** Shatadal Ghosh, Sudip Bhattacharyya, Kahkashan Rashid, Parames C. Sil

**Affiliations:** Division of Molecular Medicine, Bose Institute, P-1/12, CIT Scheme VII M, Kolkata 700054, India

**Keywords:** ALT, alanine aminotransferase, ALP, alkaline phosphatase, CAT, catalase, ERK1/2, extracellular signal regulated kinases 1/2, FRAP, ferric reducing antioxidant power, GSH, glutathione, GSSG, glutathione disulphide, GST, glutathione S-transferase, GPx, glutathione peroxidase, GR, glutathione reductase, LDH, lactate dehydrogenase, MDA, malondialdehyde, NAPQI, N-acetyl-p-benzoquinone imine, MAPK, mitogen-activated protein kinases, PSA, prostate-specific antigen, ROS, reactive oxygen species, SOD, superoxide dismutase, STZ, streptozotocin, TPTZ, 2,4,6-tripyridyl-*s*-triazine, Streptozotocin, Diabetes, Liver, Reactive oxygen species, Apoptosis, Curcumin, Antioxidant

## Abstract

Curcumin (CUR) is a highly pleiotropic molecule and possesses anti-inflammatory, hypoglycemic, antioxidative, wound-healing and antimicrobial activities. The present study was carried out to investigate whether CUR plays any beneficial role in streptozotocin (STZ) induced hepatic pathophysiology in diabetic rats. STZ exposure increased hepatic damage associated serum markers (ALT, ALP and LDH) as well as NO production in the liver tissue. Moreover, the same exposure enhanced ROS generation and lipid peroxidation; reduced GSH levels and antioxidant enzyme activities. Hyperglycemia induced hepatic pathophysiology also activated stress response pathways (involving phosphorylation of p38, ERK1/2 MAPKs and p53) and reduced mitochondrial membrane potential which in turn led to cellular apoptosis as evidenced from increased hepatic DNA fragmentation as well as FACS analysis. However, treatment with CUR effectively counteracts diabetes-induced, oxidative stress mediated hepatic damage and could act as a therapeutic in lessening liver dysfunction in diabetic subjects.

## 1 Introduction

Diabetes is the most common endocrine disorder now-a-days. It is basically a group of metabolic diseases characterized by hyperglycemia and resulting from the defects in insulin secretion, insulin action or both [1]. Hyperglycemia contributes to the progression and maintenance of overall oxidative environment. Increasing evidence from both the experimental and clinical studies indicates that oxidative stress plays a major role in the diabetic pathophysiology [2]. Reactive oxygen species (ROS) are generated disproportionately in diabetes by various pathways [3]. Among these various pathways, high glucose (and also other sugars) activates the polyol pathway, increased formation of AGEs (advanced glycation end products) along with the expression of the receptor for AGEs, activation of protein kinase C (PKC) isoforms and increased activity of the hexosamine pathway were reported to be important [4]. ROS usually damage different organs of the body by peroxidation of membrane lipids, oxidation of proteins, DNA and other intracellular macromolecules. Changes in oxidative stress biomarkers, including glutathione, superoxide dismutase, catalase, glutathione reductase, glutathione peroxidase, some vitamins and associated genes may serve as a quantitative measurement of oxidative damage in diabetes [5]. Our aim was to find out easily available and inexpensive antioxidant molecules which can effectively reduce hepatic oxidative overload under diabetic conditions. For this purpose we have chosen curcumin, a commonly used foodstuff and an important component of Indian herbal medicine. Curcumin is a diaryl heptanoid and is the principal curcuminoid of the popular South Asian spice, turmeric. It is a well-known antioxidant [6] and highly pleiotropic molecule that has been reported to exert a wide range of pharmacological activities like antibacterial [7] anti-inflammatory, anti-cancer, anti-oxidant [8], hypoglycaemic [9], [10], anti-atherosclerotic, anti-microbial [10], wound healing [11], etc. Moreover, curcumin has been found to interact directly with various intracellular signalling molecules [12]. Its ameliorative effects have been indicated to be mediated through the modulation of multiple cell signalling molecules like apoptotic proteins, cyclooxygenase (COX)-2, NF-κB [13], STAT3, IKKβ, interleukin [IL]-1β, IL-6 [14], endothelin-1, C-reactive protein (CRP) [15], GST [16], [17], PSA, pro-inflammatory cytokines (tumour necrosis factor [TNF]-α [18], VCAM, prostaglandin E2, malondialdehyde (MDA), glutathione (GSH) [19], pepsinogen, phosphorylase kinase (PhK) [20], creatinine, transferrin receptor, total cholesterol, transforming growth factor (TGF)-β, triglyceride, HO-1 [21], etc. Most importantly, curcumin has been reported to have the capacity to directly scavenge ROS [22].

We have done a thorough search in the literature related to the beneficial effects of curcumin on diabetic rat liver. All the research articles published so far focuses mainly on curcumin's beneficial roles on the biochemical parameters [23], [24]. Some recent reports also described other beneficial roles of curcumin against generalized diseased conditions; like its antioxidant effect [6], anti-diabetic effect [25], etc. Some of the structural analogues of curcumin were also investigated for the similar effects ([26]; [42]). However, detail mechanism was not carried out in any of those studies related to our experimental model. In our study, we have performed a detailed mechanistic investigation to assess not only the biochemical changes but also the molecular signalling pathways through which curcumin exert its beneficial effects. In the present study, we have investigated the protective action of curcumin, like in the enhancement of the activity of antioxidant enzymes (usually thought to be the first line of cellular defence against oxidative damage); elevation of the body weight; increase in the cellular antioxidant power (FRAP); elevation of cellular non-enzymatic antioxidant (GSH) content; increase in the mitochondrial membrane potential (Δ*ψ*_*m*_); amelioration of the tissue damage (histological assessment) and most importantly, inhibition of p53 and p38-ERK1/2 MAPKs mediated mitochondrial Bax translocation and subsequent intrinsic mitochondrial apoptotic pathway in diabetes-induced hepatic oxidative stress. The outcome of this study could help to determine the role of this bioactive molecule in reducing diabetes induced oxidative stress and might shed some light on the darkness of the gradual deterioration of this serious endocrine disorder worldwide.

## 2 Materials and methods

### 2.1 Materials

#### 2.1.1 Chemicals

Curcumin, STZ, BSA, Bradford reagent, anti-Bcl-2, anti- Bcl-XL, anti-Bad and anti-Bax antibodies were purchased from Abcam (UK). Other antibodies like anti-ERK, anti-p53, etc. were purchased from Sigma–Aldrich Chemical Company (St. Louis, MO, USA). Kits for measurement of blood glucose and LDH were purchased from Span Diagnostic Ltd., India. All other chemicals were bought from Sisco Research Laboratory, India.

#### 2.1.2 Animals

Adequate numbers of adult male Wistar rats weighing approximately 220–280 g were purchased from M/S Gosh Enterprises, Kolkata, India. All the animals were acclimatized under laboratory conditions for 2 weeks before any experiment. Animals were maintained under standard conditions of temperature (23 ± 2 °C) and humidity (50 ± 10%) with alternating 12 h light/dark cycle. The animals were given free access to tap water and fed standard pellet diet (Agro Corporation Private Ltd., Bangalore, India). All the experiments involving animals were carried out according to the guidelines of the Institutional Animal Ethical Committee (IAEC), Bose Institute, Kolkata (the permit number is IAEC/BI/3(I) cert./2010) and full details of the study was approved by both IAEC and Committee for the Purpose of Control and Supervision on Experiments on Animals (CPCSEA), Ministry of Environment & Forests, New Delhi, India (the permit number is 95/99/CPCSEA).

### 2.2 Methods

#### 2.2.1 Experimental design for in vivo treatments

Experimental design needed for the present in vivo study has been summarized as follows: Rats were randomly assigned to four groups and treated as follows:

Group 1: Normal group: rats received neither STZ nor curcumin, received vehicle only.
Group 2: CUR group: rats received only CUR (100 mg/kg body weight in olive oil) orally for 56 days (simultaneously with Group 4).
Group 3: STZ group: rats received single dose of STZ (STZ, 60 mg/kg body weight in citrate buffer, pH 4.5, i.p.) [27]. STZ-exposed rats with blood glucose level in excess of 300 mg/dL, 3 weeks after the exposure were considered as diabetic.
Group 4: STZ and CUR: post-treatment group: rats received CUR (orally, 100 mg/kg body weight in olive oil) after 3 weeks from the day on which STZ was injected until the 56th day.

In the present study, there was another group, namely, vehicle control group (STZ + olive oil). The animals in this group received STZ and after the onset of diabetes (3 weeks from the day on which STZ was injected), only olive oil was given until 56th day.

**Fig. 1:**
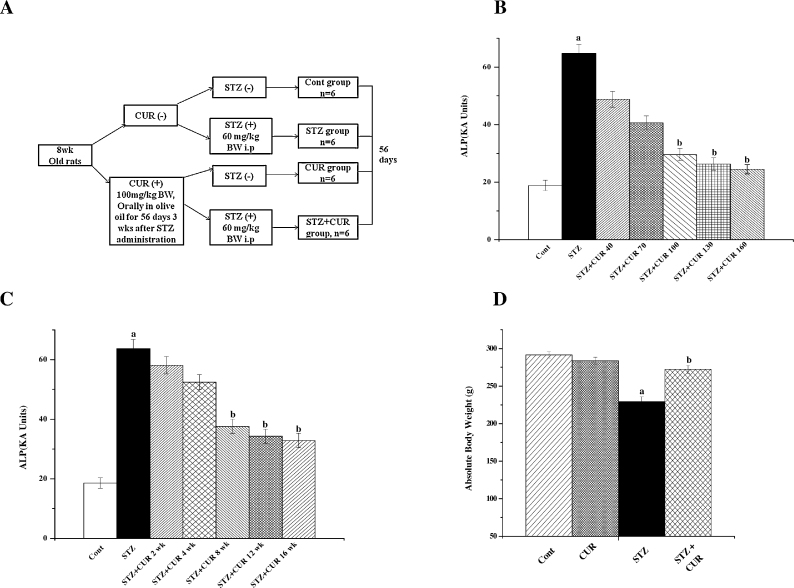
(A) Schematic diagram of the experimental protocol. (B) Representation of the dose dependent study of CUR on ALP level in STZ-treated diabetic pathophysiology in the serum of the experimental rats. Cont: measurement of serum ALP in normal rats, STZ: measurement of serum ALP in STZ administered rats, STZ + CUR 40, STZ + CUR 70, STZ + CUR 100, STZ + CUR 130 and STZ + CUR 160: measurement of serum ALP in rats which are treated with CUR at a dose of 40 mg/kg, 70 mg/kg, 100 mg/kg, 130 mg/kg and 160 mg/kg body weight, orally, respectively, after STZ administration at a dose of 60 mg/kg body weight, i.p. (C) Representation of the time dependent study of CUR on ALP activity in STZ-exposed diabetic pathophysiology in the serum of the experimental rats. Cont: measurement of serum ALP in normal rats, STZ: measurement of serum ALP in STZ exposed rats, STZ + CUR 2 wk, STZ + CUR 4 wk, STZ + CUR 8 wk, STZ + CUR 12 wk and STZ + CUR 16 wk: measurement of serum ALP in rats which are treated with CUR at a dose of 100 mg/kg body weight, orally for 2 weeks, 4 weeks, 8 weeks, 12 weeks and 16 weeks, respectively, 3 weeks after STZ administration at a dose of 60 mg/kg body weight, i.p. (D) Effects of STZ and CUR on body weight of rats. “a” indicates the significant difference between the normal control and STZ exposed groups, “b” indicates the significant difference between STZ exposed (toxin control) and CUR post-treated groups. Each column represents mean ± SEM, *n* = 6; (*p*^a^ < 0.05, *p*^b^ < 0.05).

After the experimental periods animals were sacrificed and liver was collected. The experimental design for the present study was summarized in Fig. 1A.

#### 2.2.2 Determination of dose and time-dependent activity of CUR by ALP assay

For this study, rats were randomly distributed into seven groups each consisting of six animals. The first two groups served as normal control (receiving vehicle only) and toxin control (receiving STZ at a single dose of 60 mg/kg body weight in citrate buffer, pH 4.5, i.p.), respectively. The remaining five groups of animals were treated with five different doses of CUR (40, 70, 100, 130 and 160 mg/kg body weight) for 56 days after 3 weeks followed by STZ injection (at a single dose of 60 mg/kg body weight in citrate buffer, pH 4.5, i.p.).

For the time-dependent study, rats were randomly distributed into seven groups each consisting of six animals. The first two groups served as normal control (receiving only vehicle) and toxin control (exposed to STZ at a single dose of 60 mg/kg body weight in citrate buffer, pH 4.5, i.p.), respectively. The remaining six groups of animals were treated with CUR orally at a dose of 100 mg/kg body weight, once daily for 2, 4, 8, 12 and 16 weeks after the onset of diabetes, i.e., 3 weeks after STZ administration.

#### 2.2.3 Measurement of body weight

The body weights of all the experimental animals from each group were measured.

#### 2.2.4 Preparation of liver tissue homogenate

Liver samples from experimental animals were first homogenized (1:4, w/v) in ice-cold 0.1 M phosphate buffer (pH 7.4) containing 2 mM EDTA. Then the homogenate was centrifuged at 10,000 × *g* for 30 min at 4 °C. The supernatant was collected and again centrifuged at 105,000 × *g* for 55 min. The resulting pellets called microsomal fractions were suspended in 0.25 mM sucrose solution containing 1 mM EDTA and stored at −80 °C until use. The supernatant was also collected and used for the experiments as the cytosolic fraction. The protein contents of both the cytosolic fraction and microsomal fraction were measured by the method of Bradford [28] using crystalline BSA as standard.

#### 2.2.5 Determination of hepatic markers and nitric oxide production

Specific markers related to hepatic dysfunction, e.g., ALT, AST, ALP activities and albumin were estimated by using standard kits. LDH activity was determined according to the method of Kornberg [29]. The hepatic NO level was indirectly assessed by measuring the nitrite levels in the cytosolic fraction by using a colorimetric method based on the Griess reaction [30], [31].

#### 2.2.6 Estimation of lipid peroxidation

Lipid peroxidation in terms of malondialdehyde (MDA) formation was estimated according to the method of Esterbauer et al. [32].

#### 2.2.7 Assay of cellular metabolites

GSH contents were measured, following the method of Ellman [33], using DTNB (Ellman's reagent) as the key reagent.

GSSG contents were determined according to the method of Hissin et al. [34].

#### 2.2.8 Determination of in vivo antioxidant power by FRAP assay

FRAP assay was performed according to the method as described by Benzie et al. [35].

#### 2.2.9 Assay of antioxidant enzymes

The activities of different antioxidant enzymes, SOD, CAT, GST, GR, and GPx, have been estimated in liver tissue homogenates. SOD activity was determined as described previously [36]. One unit of SOD activity is defined as the enzyme concentration that required inhibiting chromogen production by 50% in 1 min under the assay conditions.

CAT activity was estimated by following the decomposition of H_2_O_2_ at 240 nm for 10 min and was monitored spectrophotometrically, according to the method of Bonaventura et al. [37]. One unit of CAT activity is described as the amount of enzyme, which reduces one μmol of H_2_O_2_ per minute.

GST activity was assayed based on the conjugation reaction with GSH in the primary step of mercapturic acid synthesis [38]. GST activity was expressed as μmoles of CDNB conjugate formed/min/mg protein.

GR activity was determined following the method of [39].

GPx activity was measured according to the method of Flohe and Gunzler [40] using H_2_O_2_ and NADPH as substrates.

#### 2.2.10 Detection of cell death pathway by flow cytometry

Hepatocytes were isolated from all the experimental groups of rats. The animals were anaesthetized and livers were extensively perfused in phosphate buffer saline to get rid of blood and irrigated in a buffer [Hepes (10 mM), KCl (3 mM), NaCl (130 mM), NaH_2_PO_4_–H_2_O (1 mM) and glucose (10 mM)], pH 7.4 and incubated with a second buffer containing CaCl_2_ (5 mM), 0.05% collagenase type I and mixed with the buffer (previously described) for ∼45 min at 37 °C. The liver sample was then passed through a wide bore syringe, filtered, centrifuged and the pellet was suspended in PBS. Cells were then washed with PBS, centrifuged at 800 × *g* for 6 min, resuspended in ice-cold 70% ethanol/PBS, centrifuged at 800 × *g* for a further 6 min and resuspended in PBS. Cells so obtained were then incubated with propidium iodide (PI) and FITC-labelled Annexin V for 30 min at 37 °C. Excess PI and Annexin V were then washed off. Cells were then fixed and stained and finally analysed by flow cytometry using FACS Calibur (Becton Dickinson, Mountain View, CA) equipped with 488 nm argon laser light source; 515 nm band pass filter for FITC-fluorescence and 623 nm band pass filter for PI-fluorescence using CellQuest software. A scatter plot of PI-fluorescence (*y*-axis) vs FITC-fluorescence (*x*-axis) was prepared to show the effects.

#### 2.2.11 DNA fragmentation assay

Hepatic tissue was washed with STE buffer (0.1 M NaCl, 10 mM Tris–HCl, 1 mM EDTA, pH 8) and 1 mL homogenization buffer (0.1 M NaCl, 0.2 M sucrose, 0.01 M EDTA, 0.3 M Tris, pH 8), and 100 μL 10% SDS was added and mixed well by vortexing and incubated at 65 °C for 1 h. 175 μL of 8(M) potassium acetate was then added and incubated in ice-bath for 1 h, then centrifuged and the supernatant was collected. Equal volume of phenol–chloroform solution was added, mixed thoroughly and centrifuged to separate phases. The upper-most layer was taken in a fresh centrifuge tube. Equal volume of chloroform was added and centrifuged. The aqueous layer was taken in a fresh tube. 1/10th vol. of 3(M) sodium acetate (pH 7.4) and 2.5 times the volume of ethanol were added and centrifuged. The precipitated DNA was washed with 80% ethanol. The DNA fragmentation was assayed by electrophoresing genomic DNA samples on agarose/EtBr gel.

#### 2.2.12 Determination of mitochondrial membrane potential (Δ*ψ*_*m*_)

Mitochondria were isolated following the method of Hodarnau et al. [41]. The membrane potential (Δ*ψ*_*m*_) was measured using a FACS scan flow cytometer with an argon laser excitation at 488 and 525 nm band pass filter. The evaluation of the mitochondrial membrane potential (Δ*ψ*_*m*_) was determined on the basis of cell preservation of the fluorescent probe JC-1.

#### 2.2.13 Immunoblotting

Proteins (50 μg) from each sample were separated by 10% SDS-PAGE and transferred into PVDF membranes. Membranes were then blocked using BSA and incubated separately with primary antibodies of anti caspase-3, anti-p53, anti-PARP and anti-Bcl-xL (1:1000 dilution), anti cytochrome *c* (1:1000 dilution), anti Bad (1:1000 dilution), anti Bax (1:1000 dilution), anti Bcl-2 (1:1000 dilution), anti p-38 (1:1000 dilution) and anti ERK1/2 (1:1000 dilution) at 4 °C for overnight. The membranes were washed in TBST (50 mmol/L Tris–HCl, pH 7.6, 150 mmol/L NaCl, 0.1% Tween 20) for ∼30 min and incubated with appropriate HRP conjugated secondary antibody (1:2000 dilution) for 2 h at room temperature and developed by the HRP substrate 3,3′-diaminobenzidine tetrahydrochloride (DAB) system (Bangalore, India).

#### 2.2.14 Histological studies

Livers from the normal and experimental rats were fixed in 10% buffered formalin and were processed for paraffin sectioning. Sections of about 5 μm thickness were stained with haematoxylin and eosin to evaluate under light microscope. Mean values were calculated from each of six glomeruli per section.

#### 2.2.15 Statistical analysis

All experimental values have been represented as mean ± SEM (*n* = 6). Data on biochemical investigation were analysed using analysis of variance (ANOVA) and the group means were compared by Tukey's test. *p*-Values of 0.05 or less were considered significant.

## 3 Results and discussions

### 3.1 STZ induced diabetic models

STZ induced experimental diabetes is an example of chemically induced diabetes model. This model has some advantages [45] like selective loss of pancreatic beta cells leaving other pancreatic cells (alpha and delta) intact; less ketosis and related mortality. Besides, residual insulin secretion permits the animals to live long period of time without insulin treatment. In addition, this model is relatively cost effective, easy to develop and maintain. In spite of these advantages, there are some limitations [45] also. Firstly, hyperglycaemia is developed primarily by the direct cytotoxic effects of STZ on the beta cells and insulin deficiency rather than consequences of insulin resistance, i.e., it is hard to extrapolate the outcomes from any study to type II diabetes. Moreover, STZ induced diabetes has been observed to be less stable and reversible in many cases; mainly because of the spontaneous regeneration of beta cells. Hence, we had to check the blood glucose levels periodically during the experiments. In addition, STZ may produce some toxic actions on other body organs as well in spite of its cytotoxic action on beta cells. There may be another problem of variability of results in this model.

While the single high dose injection of STZ can produce type I diabetes in adult rats, STZ, when injected at the neonatal stage or immediately after birth, can lead to type II diabetes at the adult age. The neonatal STZ exposed rats are considered ([43], [44]) to be better model for the elucidation of the mechanisms associated with regeneration of the beta cells, the functional exhaustion of the beta cells and the emergence of defects in insulin action.

Recently, a novel type 2 diabetic rat model ([46]) has been introduced by the combination of short term high fat diet (HFD) feeding followed by low dose of STZ (35 mg/kg, i.p.) treatment. It is unique as the dose of STZ selected causes diabetes only in HFD-fed insulin resistant rats where as it fails to induce the same in normal control rats reflecting the actual situation in humans with risk factors of obesity and insulin resistance.

### 3.2 Dose and time-dependent effect of CUR on STZ-induced hepatotoxicity

STZ induces experimental diabetes in rats [47], thereby produces hepatotoxicity and elevates the activities of serum marker enzymes (ALT, ALP, etc.). So, we performed a dose-dependent study using ALP assay as an index of STZ mediated hepatic damage to determine the optimum dose and time for CUR treatment. The result of our study suggests that STZ at a dose of 60 mg/kg body weight up-regulated the ALP activity in serum although that could be reversed with the treatment of CUR up to a dose of 100 mg/kg body weight (Fig. 1B and C). Similarly time dependent study provided the optimum time of 8 weeks (56 days) for CUR treatment for maximum beneficial effect against STZ exposure. Effect of CUR was not much beyond this concentration or time period respectively. Therefore, after 3 weeks of STZ administration, 100 mg/kg body weight of CUR for 56 days (once, daily) was chosen as the optimum dose and time for the post-treatment study.

The beneficial effects of curcumin are mainly limited due to its poor pharmacokinetics and pharmacodynamics, i.e., short half-life, poor absorption and rapid metabolism in the GI tract. For this reason, daily intake level of 0.1–3 mg/kg-BW of curcumin has been considered as an acceptable dose by the Joint FAO/WHO Expert Committee on Food Additives, 1996 [48]. Moreover, turmeric is Generally Recognized As Safe (GRAS) by the US FDA.

Moreover, the doses administered in clinical trials are generally expected to be rather higher than those normally consumed in the diet. A good number of studies have been performed as well. In such a preclinical study involving the administration of 2% dietary curcumin (approx. 1200 mg/kg-BW) to rats for 14 days [16] or in a study involving the administration of 0.2% dietary curcumin (approx. 300 mg/kg-BW) to mice for 14 weeks, no toxicity was observed [49].

A study on the uptake and bio-distribution of dietary curcumin in rodents suggests that after ingestion orally, peak serum levels of curcumin are low [50]. In these studies, the dose of 2 g/kg resulted in 1.35 μg/mL peak serum levels [51]. In humans, a daily dose of 4–8 g resulted in peak serum levels of 0.4–3.6 μM after 1 h. In India, where the average intake of turmeric may reach the level of 2000–2500 mg per day (corresponding to approx. up to 100 mg of curcumin), no toxicities or adverse effects have been reported at the population level [52]. In another study in Taiwan, Cheng *et al.*, showed that the administration of high-dose of oral curcumin (500, 1000, 2000, 4000, and 8000 mg of curcumin daily) for 3 months showed no noticeable adverse effects [53]. Phase I clinical trials have demonstrated that curcumin is safe even at a dose of 12 g/day in humans but exhibit relatively poor bioavailability [50].

To overcome this problem associated with bioavailability of curcumin, numerous approaches have been undertaken. These approaches include the use of adjuvant like piperine (interferes with glucuronidation); the use of liposomal curcumin; administration of curcumin nanoparticles and the use of curcumin phospholipid complex, etc. [54], [55], [56]. Moreover, no significant difference was observed between the results obtained from “STZ” group and “STZ + olive oil” (vehicle control) group (data not shown).

### 3.3 Body weight

Our studies showed physical lethargy in the STZ exposed rats in the early days of the experiment and these animals gained less body weight compared to controls (Fig. 1D). CUR treatment, however, given protection against this type of deficiency suggesting the growth-inhibiting effect of STZ could be blocked by the beneficial effect of CUR.

### 3.4 Effect of CUR on biochemical parameters

#### 3.4.1 Effect on ALT, ALP, AST, albumin, LDH and NO levels

Single oral dose of STZ (60 mg/kg body weight) induced diabetes which significantly increased liver damage, as evidenced by a dramatic elevation of serum ALT, AST, ALP and LDH activities in STZ exposed group (Table 1). Results suggested that transport function and membrane permeability have been altered due to the liver injury, leading to leakage of these enzymes. The activities of those hepatotoxicity markers in CUR treated group remained almost the same as normal. In the CUR post treated group, those markers’ activities were significantly decreased towards normal, i.e., CUR protected liver (hepatocytes) from diabetic pathophysiology. The level of albumin was also decreased in STZ exposed group and shifted towards the normal value by CUR post treatment.

**Table 1:** Liver biomarker-enzyme activities.

Parameters	NOR	CUR	STZ	STZ + CUR
LDH^d^	175.99 ± 1.89	198.37 ± 2.12	467.58 ± 3.58^a^	253.51 ± 2.73^b^
ALP^e^	15.79 ± 1.35	18.37 ± 1.93	42.69 ± 2.47^a^	24.25 ± 3.01^b^
ALT^f^	60.92 ± 1.41	69.54 ± 2.14	148.75 ± 2.17^a^	81.02 ± 2.43^b^
AST^f^	142.31 ± 4.13	159.67 ± 3.82	353.33 ± 6.50^a^	215.86 ± 7.69^b^
Albumin^g^	3.55 ± 0.07	3.46 ± 0.08	2.25 ± 0.10^a^	3.10 ± 0.09^b^
Blood glucose^h^	105 ± 9	98 ± 7	385 ± 13^a^	203 ± 14^b^

a “a” values differs significantly from normal control (*p*^a^ < 0.05).

b “b” values differs significantly from toxin control (*p*^b^ < 0.05). Values are expressed as mean ± SEM, for 6 animals in each group.

d Unit/L.

e KA unit.

f IU/L.

g g/dL.

h mg/dL.

NO level was also increased significantly in STZ exposed animals (Table 2). As STZ is a nitric oxide (NO) donor and NO has been found to bring about the destruction of pancreatic islet cells, it is assumed that this molecule contributes to STZ-induced DNA damage [57], [58]. The participation of NO in exerting the cytotoxic effect of STZ was confirmed in other experiments also [59]. Moreover, increased NO production as a result of the diabetic pathophysiology was responsible for both cell apoptosis and necrosis when NO interacts with superoxide anion to form peroxynitrite. However, post-treatment with CUR effectively reduced these alterations and ameliorated hepatocytes from diabetic pathophysiology as evidenced from the results.

**Table 2:** Antioxidant-enzyme activities.

Parameters	NOR	CUR	STZ	STZ + CUR
SOD^c^	178.94 ± 5.87	164.65 ± 6.20	131.43 ± 6.06^a^	159.45 ± 7.01^b^
CAT^d^	131.89 ± 3.34	122.77 ± 4.25	68.35 ± 4.57^a^	112.04 ± 6.41^b^
GST^e^	3.29 ± 0.12	2.96 ± 0.04	1.16 ± 0.01^a^	2.59 ± 0.08^b^
GR^f^	120.57 ± 2.33	111.24 ± 3.35	65.33 ± 3.58^a^	103.93 ± 5.74^b^
GPx^g^	130.64 ± 4.84	122.47 ± 4.92	54.57 ± 3.85^a^	106.20 ± 5.71^b^
MDA^h^	1.15 ± 0.04	1.53 ± 0.05	4.05 ± 0.06^a^	2.17 ± 0.04^b^
NO production^i^	37.15 ± 1.04	42.53 ± 1.43	123.37 ± 2.63^a^	47.67 ± 1.29^b^

a “a” values differs significantly from normal control (*p*^a^ < 0.05).

b “b” values differs significantly from toxin control (*p*^b^ < 0.05).

c Unit/mg protein.

d μmol/min/mg protein.

e μmol/min/mg protein.

f nmol/min/mg protein.

g nmol/min/mg protein.

h nmol/mg protein.

i mM.

#### 3.4.2 Effect on lipid peroxidation

Lipid peroxidation gives an indication of cellular injury mediated by reactive oxygen species [60] with the concomitant destruction of membrane lipids and thereby production of lipid peroxides. The present study showed that STZ exposure significantly enhanced hepatic lipid peroxidation in the experimental animals compared to normal and only CUR treated animals. It was also seen that CUR administration significantly inhibited hepatic lipid peroxidation (Table 2).

#### 3.4.3 Effect on cellular GSH and GSSG levels

**Fig. 2:**
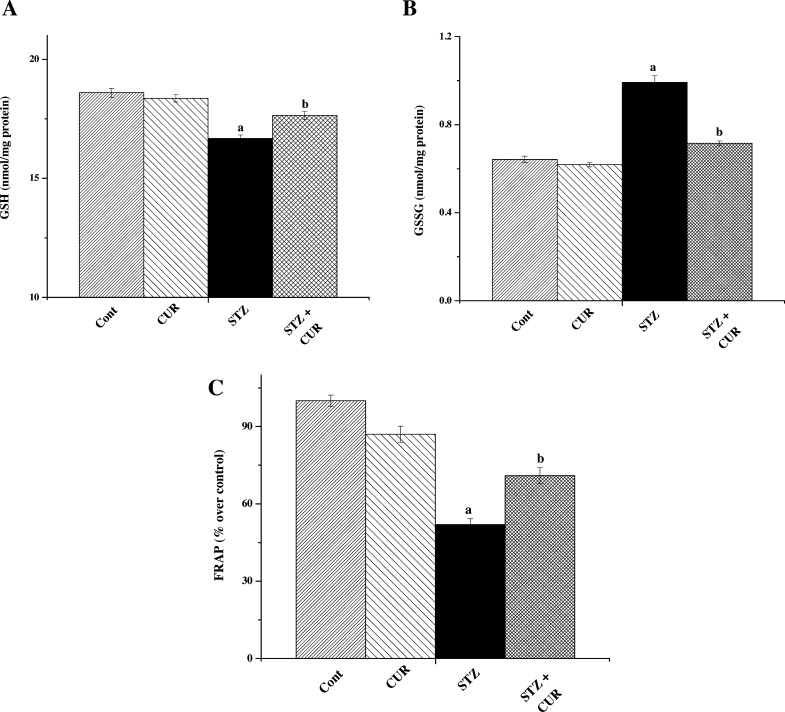
(A) Measurement of cellular levels of GSH. (B) Measurement of cellular levels of GSSG. (C) Measurement of FRAP values. “a” indicates the significant difference between the normal control and STZ exposed groups, “b” indicates the significant difference between STZ exposed (toxin control) and CUR post-treated groups. Each column represents mean ± SEM, *n* = 6 (*p*^a^ < 0.05, *p*^b^ < 0.05).

Glutathione (GSH) is a cysteine-containing peptide found in almost all forms of aerobic life [61]. It is synthesized within the cells from its constituent amino acids. GSH shows antioxidant activities due to the presence of a thiol group in its cysteine moiety; acting as a reducing agent that can be reversibly oxidized and reduced [62]. GSH plays an important role in maintaining cellular antioxidant capacity. So we measured GSH levels and found that STZ exposure resulted in rapid depletion of hepatic GSH, thereby decreasing the GSH/GSSG ratio in case of STZ group compared to normal. GSH levels remained almost the same as normal in the only CUR treated group as expected (Fig. 2A and B).

#### 3.4.4 Effect on ferric reducing antioxidant power (FRAP)

Ferric reducing antioxidant power (FRAP) assay is performed to determine the antioxidant capacity [35]. We have performed FRAP assay to evaluate the antioxidant capacity of the hepatic tissues of different groups and observed that STZ induced diabetic pathophysiology also caused a significant reduction in FRAP value (Table 1) in STZ exposed group compared to normal and only CUR treated group. However, CUR treatment restored the FRAP value towards normal (Fig. 2C).

#### 3.4.5 Effect on cellular antioxidant enzymes

Cellular antioxidant enzymes are one of the most directly acting molecules that counteract oxidative burst no matter how that is generated in the system. So we have also examined the activities of antioxidant enzymes like SOD, CAT, GST, GR and GPx in the present study. Results showed that STZ decreased the antioxidant enzyme activities in the hepatic tissues (Table 2) and CUR administration restored those enzyme activities towards the normal value. Together these results imply that CUR protected rat liver against STZ-induced diabetic pathophysiology mainly by exerting its antioxidant properties.

### 3.5 STZ-induced hepatocellular apoptosis

**Fig. 3:**
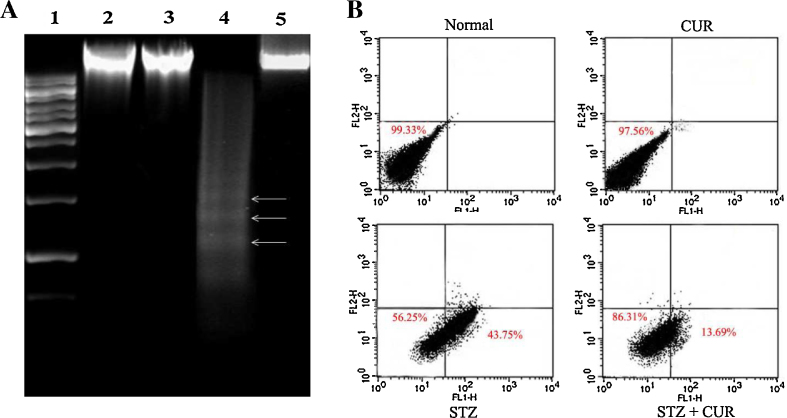
(A) DNA fragmentation pattern on agarose/EtBr gel. Lane 1 marker (1 kb DNA ladder), lanes 2, 3, 4 and 5 DNA isolated from normal, CUR treated, STZ administered, CUR post treated rats, respectively. Arrows indicate ladder formation. CUR decreased all the STZ-induced pro-apoptotic events in hepatic tissue. (B) Measurement of apoptosis in hepatocytes by the use of flow cytometry analysis. Percent distribution of apoptotic and necrotic hepatocytes shown. Cell distribution analysed using Annexin V binding and PI uptake. The FITC and PI fluorescence measured using flow cytometer with FL-1 and FL-2 filters, respectively. Results expressed as dot plot representing as one of the six independent experiments. The measurements were performed six times.

To investigate the mode of cell death, we have done fluorescence-activated cell-sorting (FACS) and DNA gel electrophoresis to identify the nature of cellular death (necrosis and/or apoptosis) in the liver. DNA isolated from STZ-exposed rats showed mainly ladder (a hallmark of apoptosis) on the agarose gel (Fig. 3A). Again, from flow-cytometric data we found that, hepatocytes isolated from STZ-exposed rats showed maximum Annexin V-FITC-binding compared to control untreated hepatocytes, but very little PI staining, indicating the majority of cells underwent apoptosis (Fig. 3B). Therefore, cross-checked with Annexin V/PI staining also support the apoptotic cell death in this pathophysiology. However, CUR administration showed a satisfactory level of improvement in hepatocytes viability in both the cases, there by suggesting the protective efficacy along with the anti-apoptotic nature of CUR.

### 3.6 Effect of CUR on hepatic histology

**Fig. 4:**
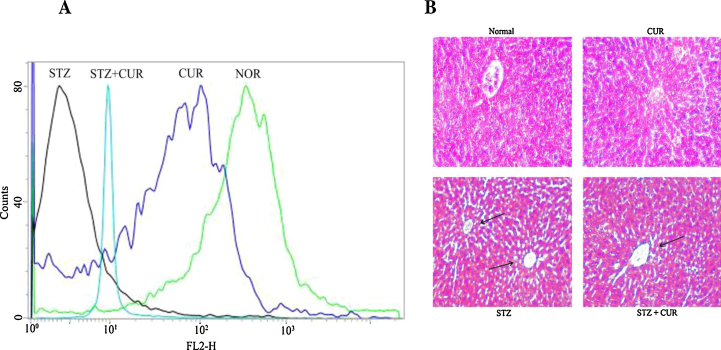
(A) Measurement of the mitochondrial membrane potential by flow cytometry analysis (using JC-1) from liver tissue homogenates. (B) Studies on histological assessments; haematoxylin and eosin stained liver section of (A) normal rat liver (×100), (B) CUR treated liver section (×100), (C) STZ intoxicated liver section (×100) and (D) CUR treated after STZ intoxicated liver section (×100). Arrows indicate centrilobular apoptosis in the liver tissue compared to the normal liver section.

Histological assessments of different liver segments of the normal and experimentally treated animals have been presented in (Fig. 4B). STZ administration induced liver tissue damage along the central vein and disorganized the normal radiating pattern of cell plates around it. However, CUR treatment showed a considerable improvement in liver morphology.

### 3.7 Effect of CUR on molecular signalling pathways

Our next goal in the present study was to explore the plausible molecular signalling pathways through which CUR exerted its ameliorative actions in diabetes-induced hepatic pathophysiology.

#### 3.7.1 Effect on p53 activation

**Fig. 5:**
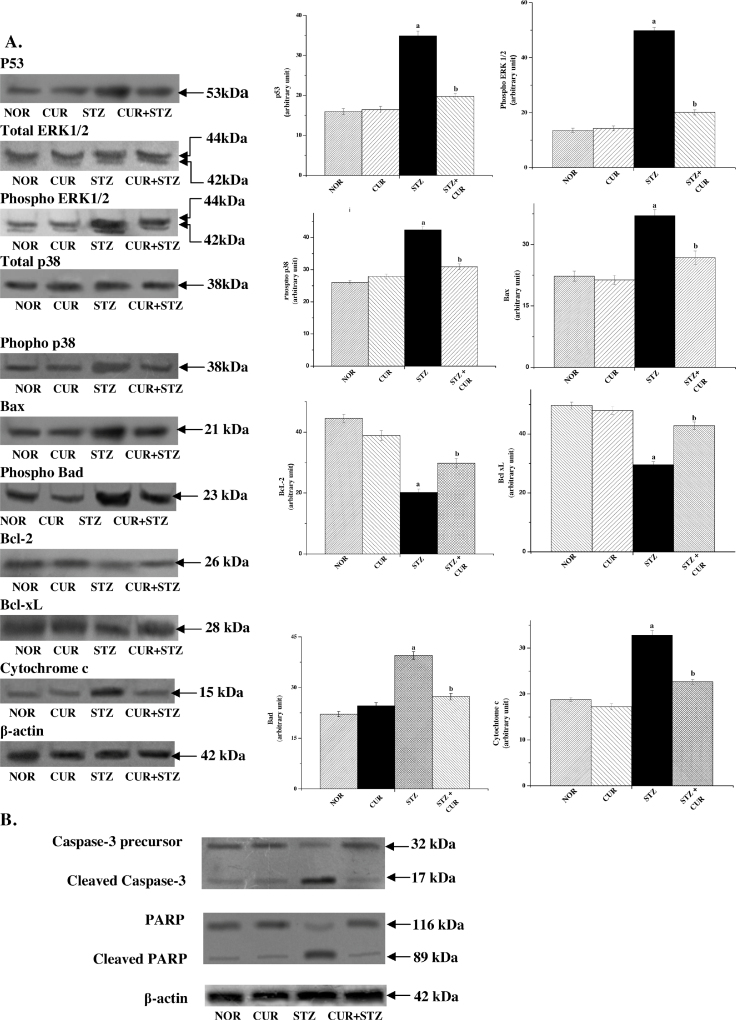
(A) Western blot analysis of different proteins such as p53, total and phospho ERK1/2, total and phospho p38, Bax, Bcl-2, Bcl-xL and cytochrome c. β-Actin served as a loading control. “a” indicates the significant difference between the normal and STZ-exposed rat liver tissue homogenates, “b” indicates the significant difference between STZ and CUR + STZ-treated liver tissue homogenates. Each column represents mean ± SEM, *n* = 6 (*p*^a^ < 0.05, *p*^b^ < 0.05). (B) Western blot analysis depicting the ultimate effects of CUR on STZ exposed mouse liver; caspase-3 activation and PARP cleavage in rat liver. β-Actin served as a loading control.

The tumour suppressor protein p53 is a stress-responsive protein and it plays a critical role in regulating both cell survival and death depending on the cell type and nature of stress involved [63]. A number of earlier studies reported that p53 plays a central role in diabetic pathophysiology in various organs like heart [1], [64], [65], pancreas [66], kidney [67], [68], etc. So, in this study we wanted to investigate the possible role of p53 in hepatic tissue and measured the protein level using immunoblot assay. We found that STZ exposure significantly increased p53 protein levels (Fig. 5A) and CUR attenuated this activation. So it can be concluded that STZ exposure could effectively induce p53 activation, and subsequent hepatic cellular death in a p53-dependent manner which is ameliorated by using CUR.

#### 3.7.2 Effect on MAPKs activation

Earlier reports suggest that oxidative stress induces MAPK family proteins [69]. Among the members of this protein family, p38 and ERK1/2 MAPKs play crucial roles in STZ-induced cell death pathways [70]. Besides, these proteins also play important roles in maintaining nuclear response to stress and cell survival [71]. So, we investigated whether p38 and ERK1/2 MAPKs have any role in the present scenario of organ pathophysiology. Results of immunoblot analysis showed that STZ-exposure did stimulate p38 and ERK1/2 MAPKs phosphorylation (Fig. 5A) in the hepatic tissue and this effect was significantly reversed by CUR treatment, suggesting that CUR attenuates STZ-induced cell death by inhibiting MAPKs activation.

In healthy cells, Bax, a proapoptotic member of the Bcl-2 protein family, is present in the cytosol. Under stressing condition, Bax undergoes a conformational change and translocated into the mitochondria from cytosol and causes damages to the outer mitochondrial membrane causing mitochondrial membrane depolarisation and subsequent release of cytochrome c into the cytosol. These sequences of events were experimentally observed in our present study by immunoblot assay of cytochrome c (Fig. 5A). Although earlier reports [72], [73] suggest that p53 stimulates the mitochondrial cell death pathway, our findings in this study clearly suggest that p38 and ERK1/2 MAPKs also initiate the same apoptotic pathway.

#### 3.7.3 Effect on mitochondrial membrane potential (Δ*ψ*_*m*_)

Maintenance of mitochondrial membrane potential (Δ*ψ*_*m*_) is fundamental for cellular survival and loss of it (Δ*ψ*_*m*_) may induce a cascade of reactions leading to cellular apoptosis [74]. Disruption of mitochondrial membrane potential (Δ*ψ*_*m*_) induces the release of cytochrome c into the cytosol and activates downstream apoptotic signalling pathways [75]. Results of our present study showed that STZ induced diabetic pathophysiology reduced the mitochondrial membrane potential significantly and CUR could ameliorate this event effectively (Fig. 4A). Our immunoblotting studies showed that cytosolic cytochrome c level was increased in STZ exposed group compared to normal as in the case of (Fig. 5A) the translocation of Bax from cytoplasm to mitochondria. In both the cases immunoblot assay also showed that CUR post treatment could effectively ameliorate these two apoptotic phenomena.

#### 3.7.4 Effect on caspase 3 activation and PARP cleavage

Following the release into the cytosol, cytochrome c induces the formation of apoptosomes which in turn activates caspase 9 and other downstream caspases [76]. In our study, occurrence of apoptosis was determined by caspase-3 activation and PARP cleavage. The cellular level of cleaved caspase 3, a product and indicator of caspase-3 activation and subsequent apoptosis [77], was significantly increased in STZ-exposed hepatocytes; however, normalcy was restored in CUR post-treated group. As expected, only CUR group showed no such indication of caspase 3 activation. We have also measured the level of PARP, the substrate of cleaved caspase 3. Immunoblot study showed significant enhancement of PARP (116 kDa) cleavage in STZ exposed group into the 89 kDa fragment although there was almost no such PARP cleavage observed in any other group (Fig. 5B).

In short, these results indicate that CUR might be a protective dietary antioxidant molecule that could effectively ameliorate all the adverse effects of STZ-induced hepatic pathophysiology by inhibiting p53 and p38-ERK1/2 MAPKs mediated mitochondrial Bax translocation and subsequent intrinsic mitochondrial apoptotic pathway.

#### 3.7.5 Effect on pro and anti-apoptotic protein levels

To further confirm our findings we have also measured the expression levels of anti-apoptotic proteins Bcl-2 and Bcl-xL and pro-apoptotic protein Bad. Both type of the proteins (pro and anti) together control the antiapoptotic–apoptotic nature of cells. In our study we observed (Fig. 5A) a marked decrease in the expression level of the Bcl-2 and Bcl-xL in the STZ exposed group, whereas no such decrease was observed in the CUR treated group or normal. The expression level of Bad, however, showed opposite phenomenon.

## 4 Conclusion

In summary, our study indicates that STZ induces diabetic pathophysiology and thereby generation of oxidative stress that leads to systematic apoptosis within liver via the activation of p38-ERK1/2 and p53-mediated molecular signalling pathways. In this circumstance, CUR, a well-known antioxidant, might protect liver (hepatocytes) primarily by two ways: firstly, it offers protection by elevating the antioxidant enzyme activities and scavenging ROS as evident from biochemical results and secondly, it plays the same ameliorative role by the inhibition of phosphorylations of p38, ERK1/2 MAPKs, subsequent Bax translocation to mitochondria and mitochondrial permeabilization. So we are hopeful about the promising solution of diabetes-induced liver tissue apoptosis using curcumin having no known adverse effect so far.

## Conflict of interest

The authors have declared that no conflict of interest exists.

## Transparency document

Transparency document

## References

[1] Ghosh J., Das J., Manna P., Sil P.C. (2011). The protective role of arjunolic acid against doxorubicin induced intracellular ROS dependent JNK-p38 and p53-mediated cardiac apoptosis. Biomaterials.

[2] Das J., Roy A., Sil P.C. (2012). Mechanism of the protective action of taurine in toxin and drug induced organ pathophysiology and diabetic complications: a review. Food Funct.

[3] Maritim A.C., Sanders R.A., Watkins J.B. (2003). Diabetes, oxidative stress, and antioxidants: a review. J. Biochem. Mol. Toxicol..

[4] Giacco F., Brownlee M. (2010). Oxidative stress and diabetic complications. Circulation research.

[5] Das J., Sil P.C. (2012). Taurine ameliorates alloxan-induced diabetic renal injury, oxidative stress-related signaling pathways and apoptosis in rats. Amino Acids.

[6] Lin J., Zheng S., Chen A. (2009). Curcumin attenuates the effects of insulin on stimulating hepatic stellate cell activation by interrupting insulin signaling and attenuating oxidative stress. Lab. Invest..

[7] Schraufstatter E., Bernt H. (1949). Antibacterial action of curcumin and related compounds. Nature.

[8] Sharma O.P. (1976). Antioxidant activity of curcumin and related compounds. Biochem. Pharmacol..

[9] Sharma S., Kulkarni S.K., Chopra K. (2006). Curcumin, the active principle of turmeric (*Curcuma longa*), ameliorates diabetic nephropathy in rats. Clin. Exp. Pharmacol. Physiol..

[10] Nishiyama T., Mae T., Kishida H., Tsukagawa M., Mimaki Y., Kuroda M., Sashida Y., Takahashi K., Kawada T., Nakagawa K., Kitahara M. (2005). Curcuminoids and sesquiterpenoids in turmeric (*Curcuma longa* L.) suppress an increase in blood glucose level in type 2 diabetic KK-Ay mice. J. Agric. Food Chem..

[11] Aggarwal B.B., Sung B. (2009). Pharmacological basis for the role of curcumin in chronic diseases: an age-old spice with modern targets. Trends Pharmacol. Sci..

[12] Gupta S.C., Prasad S., Kim J.H., Patchva S., Webb L.J., Priyadarsini I.K., Aggarwal B.B. (2011). Multitargeting by curcumin as revealed by molecular interaction studies. Nat. Prod. Rep..

[13] Dhillon N., Aggarwal B.B., Newman R.A., Wolff R.A., Kunnumakkara A.B., Abbruzzese J.L., Ng C.S., Badmaev V., Kurzrock R. (2008). Phase II trial of curcumin in patients with advanced pancreatic cancer. Clin. Cancer Res..

[14] Kim S.G., Veena M.S., Basak S.K., Han E., Tajima T., Gjertson D.W., Starr J., Eidelman O., Pollard H.B., Srivastava M., Srivatsan E.S., Wang M.B. (2011). Curcumin treatment suppresses IKKbeta kinase activity of salivary cells of patients with head and neck cancer: a pilot study. Clin. Cancer Res..

[15] Holt P.R., Katz S., Kirshoff R. (2005). Curcumin therapy in inflammatory bowel disease: a pilot study. Dig. Dis. Sci..

[16] Sharma R.A., Ireson C.R., Verschoyle R.D., Hill K.A., Williams M.L., Leuratti C., Manson M.M., Marnett L.J., Steward W.P., Gescher A. (2001). Effects of dietary curcumin on glutathione S-transferase and malondialdehyde-DNA adducts in rat liver and colon mucosa: relationship with drug levels. Clin. Cancer Res..

[17] Sharma R.A., McLelland H.R., Hill K.A., Ireson C.R., Euden S.A., Manson M.M., Pirmohamed M., Marnett L.J., Gescher A.J., Steward W.P. (2001). Pharmacodynamic and pharmacokinetic study of oral Curcuma extract in patients with colorectal cancer. Clin. Cancer Res..

[18] He Z.Y., Shi C.B., Wen H., Li F.L., Wang B.L., Wang J. (2011). Upregulation of p53 expression in patients with colorectal cancer by administration of curcumin. Cancer Invest..

[19] Kalpravidh R.W., Siritanaratkul N., Insain P., Charoensakdi R., Panichkul N., Hatairaktham S., Srichairatanakool S., Phisalaphong C., Rachmilewitz E., Fucharoen S. (2010). Improvement in oxidative stress and antioxidant parameters in beta-thalassemia/Hb E patients treated with curcuminoids. Clin. Biochem..

[20] Heng M.C., Song M.K., Harker J., Heng M.K. (2000). Drug-induced suppression of phosphorylase kinase activity correlates with resolution of psoriasis as assessed by clinical, histological and immunohistochemical parameters. Br. J. Dermatol..

[21] Shoskes D., Lapierre C., Cruz-Correa M., Muruve N., Rosario R., Fromkin B., Braun M., Copley J. (2005). Beneficial effects of the bioflavonoids curcumin and quercetin on early function in cadaveric renal transplantation: a randomized placebo controlled trial. Transplantation.

[22] Gupta S.C., Patchva S., Aggarwal B.B. (2013). Therapeutic roles of curcumin: lessons learned from clinical trials. AAPS J..

[23] Palma H.E., Wolkmer P., Gallio M., Correa M.M., Schmatz R., Thome G.R., Pereira L.B., Castro V.S., Pereira A.B., Bueno A., de Oliveira L.S., Rosolen D., Mann T.R., de Cecco B.S., Graca D.L., Lopes S.T., Mazzanti C.M. (2014). Oxidative stress parameters in blood, liver, and kidney of diabetic rats treated with curcumin and/or insulin. Mol. Cell. Biochem..

[24] Sun C., Zhang Y., Kong T., Li Y., Feng R., Wang G. (2013). Effects of curcumin intake on kidney and liver pathological changes in T2DM rats. Wei Sheng Yan Jiu.

[25] Abdel Aziz M.T., El-Asmar M.F., El-Ibrashy I.N., Rezq A.M., Al-Malki A.L., Wassef M.A., Fouad H.H., Ahmed H.H., Taha F.M., Hassouna A.A., Morsi H.M. (2012). Effect of novel water soluble curcumin derivative on experimental type-1 diabetes mellitus (short term study). Diabetol. Metab. Syndr..

[26] Pari L., Karthikesan K., Menon V.P. (2010). Comparative and combined effect of chlorogenic acid and tetrahydrocurcumin on antioxidant disparities in chemical induced experimental diabetes. Mol. Cell. Biochem..

[27] Ganda O.P., Rossini A.A., Like A.A. (1976). Studies on streptozotocin diabetes. Diabetes.

[28] Bradford M.M. (1976). A rapid and sensitive method for the quantitation of microgram quantities of protein utilizing the principle of protein-dye binding. Anal. Biochem..

[29] Kornberg A., Colowick S.P., Kaplan N.O. (1955). Lactic dehydrogenase of muscle. Methods in Enzymology.

[30] Green L.C., Wagner D.A., Glogowski J., Skipper P.L., Wishnok J.S., Tannenbaum S.R. (1982). Analysis of nitrate, nitrite, and [^15^N]nitrate in biological fluids. Anal. Biochem..

[31] Suresh Y., Das U.N. (2006). Differential effect of saturated, monounsaturated, and polyunsaturated fatty acids on alloxan-induced diabetes mellitus. Prostaglandins Leukot. Essent. Fatty Acids.

[32] Esterbauer H., Cheeseman K.H. (1990). Determination of aldehydic lipid peroxidation products: malonaldehyde and 4-hydroxynonenal. Methods Enzymol..

[33] Ellman G.L. (1959). Tissue sulfhydryl groups. Arch. Biochem. Biophys..

[34] Hissin P.J., Hilf R. (1976). A fluorometric method for determination of oxidized and reduced glutathione in tissues. Anal. Biochem..

[35] Benzie I.F., Strain J.J. (1999). Ferric reducing/antioxidant power assay: direct measure of total antioxidant activity of biological fluids and modified version for simultaneous measurement of total antioxidant power and ascorbic acid concentration. Methods Enzymol..

[36] Manna P., Sinha M., Sil P.C. (2008). Protection of arsenic-induced testicular oxidative stress by arjunolic acid. Redox Rep..

[37] Bonaventura J., Schroeder W.A., Fang S. (1972). Human erythrocyte catalase: an improved method of isolation and a reevaluation of reported properties. Arch. Biochem. Biophys..

[38] Habig W.H., Pabst M.J., Jakoby W.B. (1974). Glutathione S-transferases. The first enzymatic step in mercapturic acid formation. J. Biol. Chem..

[39] Smith I.K., Vierheller T.L., Thorne C.A. (1988). Assay of glutathione reductase in crude tissue homogenates using 5,5′-dithiobis(2-nitrobenzoic acid). Anal. Biochem..

[40] Flohe L., Gunzler W.A. (1984). Assays of glutathione peroxidase. Methods Enzymol..

[41] Hodarnau A., Dancea S., Barzu O. (1973). Isolation of highly purified mitochondria from rat pancreas. J. Cell Biol..

[42] Pari L., Murugan P. (2007). Antihyperlipidemic effect of curcumin and tetrahydrocurcumin in experimental type 2 diabetic rats. Ren Fail.

[43] Bonner-Weir S., Trent D.F., Honey R.N., Weir G.C. (1981). Responses of neonatal rat islets to streptozotocin: limited B-cell regeneration and hyperglycemia. Diabetes.

[44] Fernandez-Alvarez J., Barbera A., Nadal B., Barcelo-Batllori S., Piquer S., Claret M., Guinovart J.J., Gomis R. (2004). Stable and functional regeneration of pancreatic beta-cell population in nSTZ-rats treated with tungstate. Diabetologia.

[45] Srinivasan K., Ramarao P. (2007). Animal models in type 2 diabetes research: an overview. Indian J Med Res..

[46] Srinivasan K., Viswanad B., Asrat L., Kaul C.L., Ramarao P. (2005). Combination of high-fat diet-fed and low-dose streptozotocin-treated rat: a model for type 2 diabetes and pharmacological screening. Pharmacol Res..

[47] Szkudelski T. (2001). The mechanism of alloxan and streptozotocin action in B cells of the rat pancreas. Physiol. Res..

[48] (1996). Clinical development plan: curcumin. J. Cell. Biochem. Suppl..

[49] Perkins S., Verschoyle R.D., Hill K., Parveen I., Threadgill M.D., Sharma R.A., Williams M.L., Steward W.P., Gescher A.J. (2002). Chemopreventive efficacy and pharmacokinetics of curcumin in the min/+ mouse, a model of familial adenomatous polyposis. Cancer Epidemiol. Biomark. Prev..

[50] Anand P. (2007). Bioavailability of curcumin: problems and promises. Mol. Pharm..

[51] Shoba G. (1998). Influence of piperine on the pharmacokinetics of curcumin in animals and human volunteers. Planta Med..

[52] Chainani-Wu N. (2003). Safety and anti-inflammatory activity of curcumin: a component of tumeric (*Curcuma longa*). J. Altern. Complement. Med..

[53] Cheng A.L. (2001). Phase I clinical trial of curcumin, a chemopreventive agent, in patients with high-risk or pre-malignant lesions. Anticancer Res..

[54] Bisht S. (2007). Polymeric nanoparticle-encapsulated curcumin (nanocurcumin): a novel strategy for human cancer therapy. J. Nanobiotechnol..

[55] Maiti K. (2007). Curcumin-phospholipid complex: preparation, therapeutic evaluation and pharmacokinetic study in rats. Int. J. Pharm..

[56] Tiyaboonchai W., Tungpradit W., Plianbangchang P. (2007). Formulation and characterization of curcuminoids loaded solid lipid nanoparticles. Int. J. Pharm..

[57] Kroncke K.D., Fehsel K., Sommer A., Rodriguez M.L., Kolb-Bachofen V. (1995). Nitric oxide generation during cellular metabolization of the diabetogenic N-methyl-N-nitroso-urea streptozotozin contributes to islet cell DNA damage. Biol. Chem. Hoppe-Seyler.

[58] Morgan N.G., Cable H.C., Newcombe N.R., Williams G.T. (1994). Treatment of cultured pancreatic B-cells with streptozotocin induces cell death by apoptosis. Biosci. Rep..

[59] Turk J., Corbett J.A., Ramanadham S., Bohrer A., McDaniel M.L. (1993). Biochemical evidence for nitric oxide formation from streptozotocin in isolated pancreatic islets. Biochem. Biophys. Res. Commun..

[60] Marnett L.J. (1999). Lipid peroxidation-DNA damage by malondialdehyde. Mutat. Res..

[61] Meister A., Anderson M.E. (1983). Glutathione. Annu. Rev. Biochem..

[62] Meister A. (1988). Glutathione metabolism and its selective modification. J. Biol. Chem..

[63] Nithipongvanitch R., Ittarat W., Velez J.M., Zhao R., St Clair D.K., Oberley T.D. (2007). Evidence for p53 as guardian of the cardiomyocyte mitochondrial genome following acute adriamycin treatment. J. Histochem. Cytochem..

[64] Nakamura H., Matoba S., Iwai-Kanai E., Kimata M., Hoshino A., Nakaoka M., Katamura M., Okawa Y., Ariyoshi M., Mita Y., Ikeda K., Okigaki M., Adachi S., Tanaka H., Takamatsu T., Matsubara H. (2012). p53 promotes cardiac dysfunction in diabetic mellitus caused by excessive mitochondrial respiration-mediated reactive oxygen species generation and lipid accumulation. Circ. Heart Fail..

[65] Jazayeri L., Callaghan M.J., Grogan R.H., Hamou C.D., Thanik V., Ingraham C.R., Capell B.C., Pelo C.R., Gurtner G.C. (2008). Diabetes increases p53-mediated apoptosis following ischemia. Plast. Reconstr. Surg..

[66] Hoshino A., Ariyoshi M., Okawa Y., Kaimoto S., Uchihashi M., Fukai K., Iwai-Kanai E., Ikeda K., Ueyama T., Ogata T., Matoba S. (2014). Inhibition of p53 preserves Parkin-mediated mitophagy and pancreatic beta-cell function in diabetes. Proc. Natl. Acad. Sci. U. S. A..

[67] Deshpande S.D., Putta S., Wang M., Lai J.Y., Bitzer M., Nelson R.G., Lanting L.L., Kato M., Natarajan R. (2013). Transforming growth factor-beta-induced cross talk between p53 and a microRNA in the pathogenesis of diabetic nephropathy. Diabetes.

[68] Singh S., Raina V., Chavali P.L., Dubash T., Kadreppa S., Parab P., Chattopadhyay S. (2012). Regulation of GAD65 expression by SMAR1 and p53 upon Streptozotocin treatment. BMC Mol. Biol..

[69] Manna P., Das J., Ghosh J., Sil P.C. (2010). Contribution of type 1 diabetes to rat liver dysfunction and cellular damage via activation of NOS, PARP, IkappaBalpha/NF-kappaB, MAPKs, and mitochondria-dependent pathways: prophylactic role of arjunolic acid. Free Radic. Biol. Med..

[70] Roux P.P., Blenis J. (2004). ERK and p38 MAPK-activated protein kinases: a family of protein kinases with diverse biological functions. Microbiol. Mol. Biol. Rev..

[71] Khatun S., Chaube S.K., Bhattacharyya C.N. (2013). p53 activation and mitochondria-mediated pathway are involved during hanging death-induced neuronal cell apoptosis in dentate gyrus region of the rat brain. Springerplus.

[72] Das J., Ghosh J., Manna P., Sil P.C. (2012). Taurine protects rat testes against doxorubicin-induced oxidative stress as well as p53, Fas and caspase 12-mediated apoptosis. Amino Acids.

[73] Lai H.C., Liu T.J., Ting C.T., Sharma P.M., Wang P.H. (2003). Insulin-like growth factor-1 prevents loss of electrochemical gradient in cardiac muscle mitochondria via activation of PI 3 kinase/Akt pathway. Mol. Cell. Endocrinol..

[74] Li P., Nijhawan D., Wang X. (2004). Mitochondrial activation of apoptosis. Cell.

[75] Li P., Nijhawan D., Budihardjo I., Srinivasula S.M., Ahmad M., Alnemri E.S., Wang X. (1997). Cytochrome c and dATP-dependent formation of Apaf-1/caspase-9 complex initiates an apoptotic protease cascade. Cell.

[76] Salvesen G.S. (2002). Caspases: opening the boxes and interpreting the arrows. Cell Death Differ..

[77] Walters J., Pop C., Scott F.L., Drag M., Swartz P., Mattos C., Salvesen G.S., Clark A.C. (2009). A constitutively active and uninhibitable caspase-3 zymogen efficiently induces apoptosis. Biochem. J..

